# Trained immunity in gout: epigenetic and metabolic reprogramming of innate immune memory

**DOI:** 10.3389/fimmu.2026.1914001

**Published:** 2026-08-12

**Authors:** Xin Chen, Yongtong Huan, Raobin Xu, Jiayi Fan, Jieru Han

**Affiliations:** 1 First Clinical Medical College, Heilongjiang University of Chinese Medicine, Harbin, China; 2 Second Clinical Medical College, Heilongjiang University of Chinese Medicine, Harbin, China; 3 School of Basic Medical Sciences, Heilongjiang University of Chinese Medicine, Harbin, China

**Keywords:** clonal haematopoiesis, epigenetics, gout, inflammation, metabolic reprogramming, monosodium urate, NLRP3 inflammasome, trained immunity

## Abstract

Gout is a chronic inflammatory arthritis driven by monosodium urate (MSU) crystal deposition. Its global prevalence is rising steadily. Only a minority of hyperuricemic individuals develop gout, and flares often recur despite controlled serum urate, pointing to mechanisms beyond simple crystal-induced inflammation. This review synthesizes evidence that trained immunity—the persistent epigenetic and metabolic reprogramming of innate immune cells—underpins these paradoxes. MSU crystals and soluble urate act as dual inducers: crystals trigger acute flares via NOD−, LRR− and pyrin domain−containing protein 3(NLRP3) and also establish long−lived myeloid memory through c−Jun N−terminal kinase (JNK)–c−Jun proto−oncogene (JUN) and mechanistic target of rapamycin (mTOR)–hypoxia−inducible factor 1−alpha (HIF-1α) axes, while soluble urate primes cells via DNA hypomethylation and histone modifications. We detail the molecular architecture of trained immunity in gout, including central [hematopoietic stem and progenitor cells (HSPC)] and peripheral training, metabolic rewiring (glycolysis, succinate), epigenetic marks (H3K4me3, H3K27ac, DNA methylation), and non−coding RNA regulation. We discuss how trained macrophages and Th17 cells form a self−amplifying loop, and how systemic trained immunity links gout to cardiovascular disease, chronic kidney disease, and metabolic syndrome, with clonal haematopoiesis of indeterminate potential (CHIP) as an age−related amplifier. Finally, we evaluate therapeutic strategies targeting epigenetic enzymes, metabolic nodes, and interleukin−1 beta (IL−1β), and highlight biomarkers and trial design needed to translate trained immunity modulation into clinical practice.

## 1 Introduction

Gout, a chronic inflammatory arthritis driven by MSU crystal deposition in joints and periarticular tissues (1–4), imposes a rapidly growing global burden—a trend fuelled largely by concurrent epidemics of obesity and metabolic syndrome (5). The canonical model appears deceptively linear: hyperuricemia promotes MSU crystallization (6), crystals activate the NLRP3 inflammasome, and the ensuing IL-1β release ignites acute joint inflammation (7). Yet this neat framework crumbles when confronted by two stubborn clinical paradoxes. First, only 10–15% of hyperuricemic individuals ever develop gout, proving that urate excess is necessary but far from sufficient for disease onset (8, 9). Second, even among those who do, recurrent flares often strike spontaneously—and unpredictably—despite serum urate levels being well-controlled by urate-lowering therapy (9). These observations have led to the hypothesis that trained immunity may explain them, but direct human evidence is still preliminary.

For this narrative review, we searched PubMed and Web of Science databases for articles published up to December 2025 using combinations of the following keywords: ‘trained immunity’, ‘trained immunity’, ‘gout’, ‘monosodium urate’, ‘epigenetics’, ‘metabolic reprogramming’, ‘NLRP3 inflammasome’, ‘clonal haematopoiesis’, and ‘innate immune memory’. We prioritised peer-reviewed original research articles, systematic reviews, and high-impact mechanistic studies. References were also identified through manual screening of bibliographies of key articles. Given the narrative scope, we focused on synthesising conceptual advances and mechanistic insights rather than performing a quantitative meta-analysis.

The specific objectives of this review are: (i) to synthesise current evidence linking trained immunity to gout pathogenesis; (ii) to delineate the molecular architecture of trained immunity in gout, including epigenetic, metabolic, and cell−autonomous mechanisms; (iii) to examine how systemic trained immunity connects gout to its comorbidities; and (iv) to critically evaluate therapeutic strategies targeting trained immunity and identify key challenges for clinical translation.

Evidence for trained immunity in gout, however, is largely indirect, coming primarily from ex vivo studies using supraphysiological urate concentrations or MSU crystal stimulation of healthy donor monocytes; these findings should be interpreted with caution until confirmed in patient−based longitudinal studies. Innate immune cells — monocytes, macrophages, and NK cells — undergo sustained reprogramming after an initial insult, resulting in amplified or blunted responses upon rechallenge (5, 10). This occurs across a spectrum: transient priming, durable training (anchored by epigenetic and metabolic rewiring), and tolerance. In gout, the balance between training and tolerance dictates disease trajectory —acute MSU-induced inflammation normally resolves via alternative activation states and Treg infiltration, but repeated crystal deposition persistently tilts this balance toward hyper-reactivity (11, 12).

To provide a clear conceptual framework, we distinguish four levels of evidence relevant to trained immunity in gout: (i) acute inflammatory responses occurring during ongoing stimulation (e.g., NLRP3 activation and IL−1β release during an acute flare); (ii) persistent inflammation due to continuing crystal exposure or sustained hyperuricaemia; (iii) epigenetic alterations observed in cross−sectional human studies, which demonstrate association but not causation; and (iv) bona fide trained immunity, defined by the operational sequence of an initial stimulus, removal of that stimulus, a defined resting interval, and a demonstrably altered response upon rechallenge. As will become evident, most of the gout−related evidence falls into categories (i)–(iii), whereas category (iv) evidence remains limited and requires further investigation.

Critically, MSU crystals are not merely passive inflammatory debris—they belong to a broader family of damage-associated molecular patterns (DAMPs), alongside HMGB1 and oxidized LDL, that actively sculpt long-term myeloid behaviour, often via the mTOR/HIF-1α or spleen tyrosine kinase(SYK) signalling axes (12, 13). It should be noted that metabolic reprogramming and epigenetic modifications are not exclusive to trained immunity—adaptive lymphocytes also undergo profound metabolic and epigenetic changes upon activation and differentiation. However, what distinguishes trained immunity is the persistence of these changes in the absence of genomic recombination and their transmission through myeloid progenitor cells, enabling functional memory that outlasts the lifespan of individual differentiated cells. This durability, rather than the metabolic or epigenetic mechanisms per se, defines the unique nature of innate immune memory and explains its relevance to chronic inflammatory conditions such as gout.

What makes gout uniquely compelling is the dual action of urate: the crystalline form ignites acute flares while simultaneously seeding long-lived innate memory (6, 7), and—even more provocatively—soluble urate itself, prior to any crystal formation, can impose persistent epigenetic changes on circulating monocytes (9). Here we synthesize evidence positioning trained immunity as a core driver in gout pathophysiology and evaluate its therapeutic implications, while acknowledging that most supportive data are mechanistic and require corroboration in clinical cohorts.

## 2 The molecular architecture of trained immunity

### 2.1 Central versus peripheral trained immunity

trained immunity operates as a dual−compartment phenomenon encompassing both peripheral training in mature innate cells and central training in hematopoietic stem and progenitor cells (HSPCs) (14). In the peripheral compartment, tissue−resident macrophages and circulating monocytes directly encounter MSU crystals, which act as endogenous DAMPs (15). Phagocytosis of crystals activates the NLRP3 inflammasome, leading to caspase−1−dependent IL−1β release—the master cytokine orchestrating acute gout flares (7). Beyond this immediate response, MSU crystals induce a trained state through metabolic and epigenetic reprogramming, resulting in amplified transcriptional responses upon subsequent challenges (16). Concurrently, systemic inflammation generated by MSU deposition, particularly IL−1β surges, reaches the bone marrow niche and engages HSPCs, initiating central trained immunity (16). These long−lived stem cells undergo durable epigenetic and metabolic changes that skew haematopoiesis toward the myeloid lineage (13). Newly generated monocytes and neutrophils inherit the ‘trained’ signature, entering circulation already primed for hyper−inflammation (17). This self−sustaining loop explains why gout patients remain susceptible to recurrent flares even during intercritical periods and why flare severity can increase over time, reflecting inflammatory memory imprinted at the stem cell level (**Table 1**) (14).

**Table 1:** Trained immunity manifests differently across innate immune cell types with important implications for gout.

Cell type	Key trained immunity features	References
Monocytes/Macrophages	Induction of aerobic glycolysis (Warburg effect) Succinate accumulation Enrichment of H3K4me3 and H3K27ac at promoters of pro-inflammatory genes (*IL1B*,*TNF*,*IL6*)	(18)
NK cells	Exhibit trained-like properties after cytokine or DAMP exposure Enhanced cytotoxicity Increased IFN-γ production	(19)
Dendritic cells	Training-induced enhancement of antigen presentation Increased cytokine secretion Bridge innate and adaptive immune responses	(20)
CD4+ T cells	HIF1α−driven glycolytic reprogramming promotes Th17 differentiation Forms a pro−inflammatory loop with trained myeloid cells Represents an extended, debated scope of trained immunity beyond classical innate lineages	(21)
Neutrophils	Short-lived but can be trained at the HSPC level in bone marrow Form neutrophil extracellular traps (NETs) NETs propagate inflammatory signals	(22)

HSPC, hematopoietic stem and progenitor cells; NK, natural killer; IFN−γ, interferon−gamma; NETs, neutrophil extracellular traps.

This stem−cell−level transmission of epigenetic and metabolic memory—rather than the presence of these modifications per se—is what fundamentally distinguishes trained immunity from the activation−associated changes seen in adaptive lymphocytes. In the peripheral compartment, tissue-resident macrophages and circulating monocytes directly encounter MSU crystals, which act as endogenous danger-associated molecular patterns (DAMPs) (15). Phagocytosis of crystals activates the NLRP3 inflammasome, leading to caspase-1-dependent IL-1β release – the master cytokine orchestrating acute gout flares (7). Beyond this immediate response, MSU crystals induce a trained state in these cells through metabolic and epigenetic reprogramming, resulting in an amplified transcriptional response upon subsequent challenges (16). It is important to note that while this central training model is supported by evidence from other inflammatory conditions, the full experimental sequence—stimulus, withdrawal, rest, rechallenge—has not been directly demonstrated for gout. The model therefore represents a working hypothesis requiring formal testing in the gout context.

### 2.2 Epigenetic mechanisms: histone modifications, DNA methylation, chromatin architecture, and their hierarchical interplay

**Figure 1:**
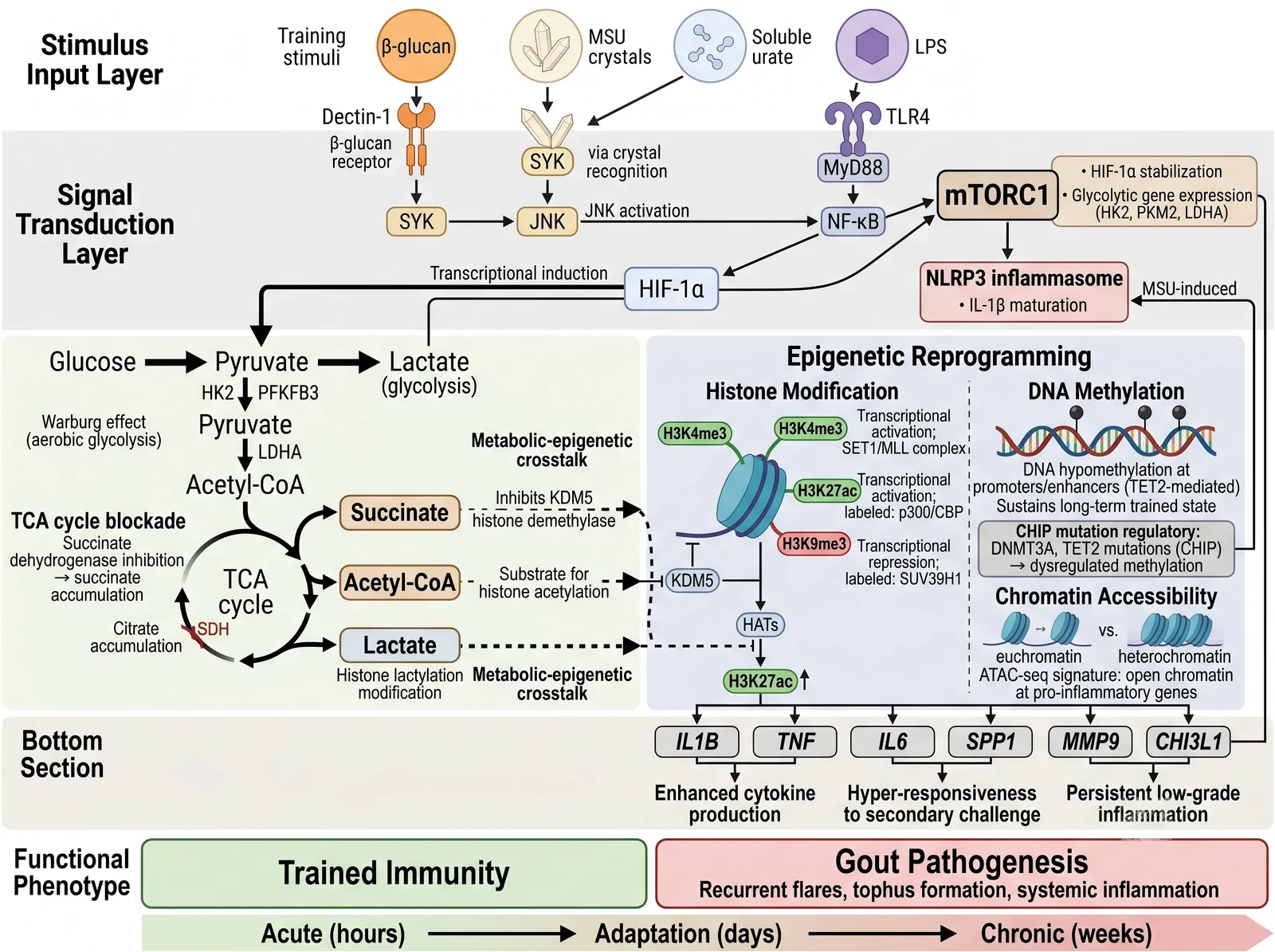
A comprehensive schematic illustrating the metabolic and epigenetic pathways underlying trained immunity. Solid arrows indicate mechanisms with direct experimental support; dashed arrows indicate indirect evidence or extrapolation; dotted arrows indicate hypotheses requiring validation in gout.

Metabolic reprogramming provides the energetic foundation for trained immunity (**Figure 1**). The shift from oxidative phosphorylation to aerobic glycolysis is an active regulatory mechanism shaping the epigenetic landscape (23). The AKT/mTOR/HIF−1α axis orchestrates this shift: upon MSU recognition, mTORC1 activation stabilises HIF−1α even under normoxia, transcriptionally upregulating glycolytic enzymes (24). Glycolytically derived metabolites fuel histone acetyltransferases for H3K27ac deposition, while succinate accumulation inhibits histone demethylases, preserving H3K4me3 at inflammatory loci and ‘locking’ cells into a hyper−responsive state (25, 26).

While H3K4me3 and H3K27ac deposition is a hallmark of the acute establishment of trained immunity, these modifications are inherently dynamic and can be reversed upon withdrawal of the training stimulus. Durable epigenetic memory—particularly that which persists through cell division—likely involves additional layers of regulation, including DNA methylation at CpG islands, changes in chromatin higher−order structure, and the recruitment of chromatin architectural proteins such as CTCF and cohesin. These more stable epigenetic marks may be particularly relevant to the chronic, relapsing nature of gout, where innate immune hyper−responsiveness persists for months to years even after serum urate normalization. This inhibition leads to persistent retention of H3K4me3 at inflammatory gene loci, “locking” cells into a hyper-responsive state (26).

DNA methylation provides a more stable epigenetic layer that may underpin long−term trained immunity in gout. Genome−wide methylation profiling has revealed widespread differential methylation in immune cells of gout patients compared with healthy controls, with enrichment in pathways related to Th17 cell differentiation and osteoclastogenesis (27). Notably, DNA methylation changes at promoters of inflammatory genes such as IL1B and NLRP3 have been associated with urate exposure, suggesting that soluble urate itself may impose durable epigenetic marks that persist even after the removal of the training stimulus (16). These observations support a model in which DNA methylation serves as a molecular ‘imprint’ of prior urate exposure, maintaining a primed state in myeloid cells and contributing to flare recurrence.

Beyond linear epigenetic marks, emerging evidence implicates changes in three−dimensional chromatin architecture—including enhancer–promoter looping and topologically associating domain (TAD) reorganization—in the maintenance of trained immunity. Such structural changes could provide a durable scaffold for sustained transcriptional memory by bringing distant regulatory elements into proximity with inflammatory gene promoters, thereby facilitating rapid transcriptional reactivation upon rechallenge. While this area remains underexplored in gout, it represents a promising frontier for understanding the persistence of innate immune memory.

Beyond classical innate immune cells, recent studies have revealed that CD4+ T lymphocytes also undergo HIF−1α−mediated metabolic reprogramming in the gouty milieu. It should be noted that classic trained immunity is strictly defined as memory properties of innate immune cells, and whether adaptive lymphocyte reprogramming falls under the trained immunity framework remains debated in the field.

Mechanistically, MSU−driven inflammation induces HIF−1α upregulation and glycolytic shift in CD4+ T cells, which promotes Th17 differentiation and IL−17A production. This forms a self−amplifying innate−adaptive crosstalk loop: epigenetically trained macrophages secrete IL−1β and IL−6 to drive Th17 polarization, and Th17−derived IL−17 in turn activates stromal cells and myeloid cells to amplify inflammation (28, 29). In this context, the AKT/mTOR/HIF−1α axis serves as a unifying metabolic target that can simultaneously modulate both innate immune training and adaptive Th17−mediated pathology in gout (25).

### 2.3 Non-coding RNAs in trained immunity

An emerging layer of regulation involves non-coding RNAs: microRNAs (miRNAs), long non-coding RNAs (lncRNAs), and circular RNAs (circRNAs) modulate trained immunity by targeting key metabolic and epigenetic regulators (30). This regulatory network adds further complexity and potential therapeutic leverage points (30).

In the context of gout, MSU crystal-induced inflammation alters multiple ncRNAs in monocytes and macrophages (31). For instance, certain microRNAs act as negative feedback regulators, while others enhance glycolytic reprogramming by targeting key signalling nodes (32–34). LncRNAs such as NEAT1 and MALAT1 scaffold chromatin-modifying complexes (35). In MSU-trained macrophages, NEAT1 promotes H3K4me3 deposition at pro-inflammatory gene promoters by sequestering repressive complexes (36). CircRNAs, highly stable due to their closed structure, may act as miRNA sponges or directly interact with transcription factors (37). For example, clinical profiling of gout patients shows that circRNA0001359 is upregulated in circulation and positively correlates with flare frequency; *in vitro* mechanistic assays further indicate it may exert pro−inflammatory effects by stabilizing HIF1α mRNA (38). Thus, ncRNAs constitute an additional post-transcriptional and epigenetic control layer, offering diagnostic and therapeutic opportunities (39).

This cell-type specificity implies that therapeutic strategies must consider distinct molecular dependencies (40). HIF−1α serves as a shared metabolic checkpoint for both trained monocytes and Th17 cells. In myeloid cells, HIF−1α drives the glycolytic reprogramming required for the metabolic and epigenetic consolidation of trained immunity. In CD4+ T cells, HIF−1α acts as a metabolic switch that promotes Th17 differentiation while suppressing Treg commitment. Thus, targeting HIF−1α simultaneously impairs both the hyper−responsive innate compartment and the Th17−mediated adaptive amplification loop, offering a dual anti−inflammatory effect. Additionally, modulating granulopoiesis may be required to control hyper-reactive neutrophils – a multi-cell perspective essential for treating chronic inflammatory diseases like gout (41).

## 3 MSU crystals and soluble urate as unique inducers of trained immunity

### 3.1 MSU crystals as a non-canonical trained immunity inducer

MSU crystals were traditionally viewed as passive DAMPs triggering NLRP3 and IL−1β. Recent work revises this view: MSU acts as an active alarmin that initiates a cell−autonomous transcriptional and metabolic reprogramming in macrophages (42). This response is distinct from canonical PAMP−driven pathways [e.g., lipopolysaccharide (LPS)–Toll−like receptor 4 (TLR4)]. Importantly, however, the evidence for this JNK–JUN axis comes from variable experimental setups, and a clear distinction must be made between studies using LPS−primed cells versus those using MSU crystals alone, as the two models capture fundamentally different aspects of the macrophage response to crystals (42, 43).

The classical two−signal model remains valid—priming (e.g., by microbial components) upregulates NLRP3 and pro−IL−1β via NF−κB (7); MSU phagocytosis disrupts lysosomes, causing K^+^ efflux and ROS, leading to inflammasome assembly and IL−1β maturation (44). However, in interpreting these findings, it is critical to distinguish between experimental models that use LPS priming prior to MSU crystals stimulation—which primarily interrogate the NLRP3 inflammasome second signal—and those that examine MSU crystals alone. The latter better reflects the initial cell−autonomous phase of crystal recognition. Using genome−wide transcriptomic analysis in non−primed human and murine macrophages, Cobo and colleagues demonstrated that MSU crystals alone induces a metabolic−inflammatory transcriptional program markedly distinct from that induced by LPS, with unique upregulation of genes involved in lipid and amino acid metabolism, glycolysis, and SLC transporters (15). Mechanistically, MSU crystals alone drives this program through persistent JNK activation and JUN binding to target gene promoters, and notably, this occurs independently of inflammasome activation (15). Extending these observations, a subsequent study identified a bifurcated transcriptional response in particle−exposed macrophages, revealing a novel AMPK–TFEB/TFE3–DNMT3A/DOT1L axis that regulates lysosomal acidification gene expression independently of the inflammatory pathway (45). These findings underscore that MSU crystals engages cell−autonomous training programs that are separable from the canonical inflammasome pathway. Beyond these inflammasome−independent effects, MSU also initiates a parallel, sustained transcriptional program triggered by the crystalline geometry of MSU, specifically the {011} crystal face, and driven by persistent JNK activation, which phosphorylates JUN (AP−1 component) and enhances binding to target gene promoters (46). This JNK–JUN axis underpins a sterile, crystal−driven form of “training” that is structurally encoded and signalling−pathway specific (46).

While the NLRP3 inflammasome–caspase−1–IL−1β axis is undoubtedly central to acute gout flares, emerging evidence indicates that MSU crystals also initiates inflammasome−independent transcriptional programmes. 15 demonstrated that the metabolic−inflammatory transcriptional program induced by MSU crystals alone is regulated through persistent JNK signalling and JUN binding to target gene promoters—a process that is independent of inflammasome activation. This observation challenges the notion that MSU crystals effects are exclusively mediated through the canonical inflammasome pathway and highlights the existence of parallel, cell−autonomous training programmes that may contribute to the chronicity of gout independently of IL−1β.

**Figure 2:**
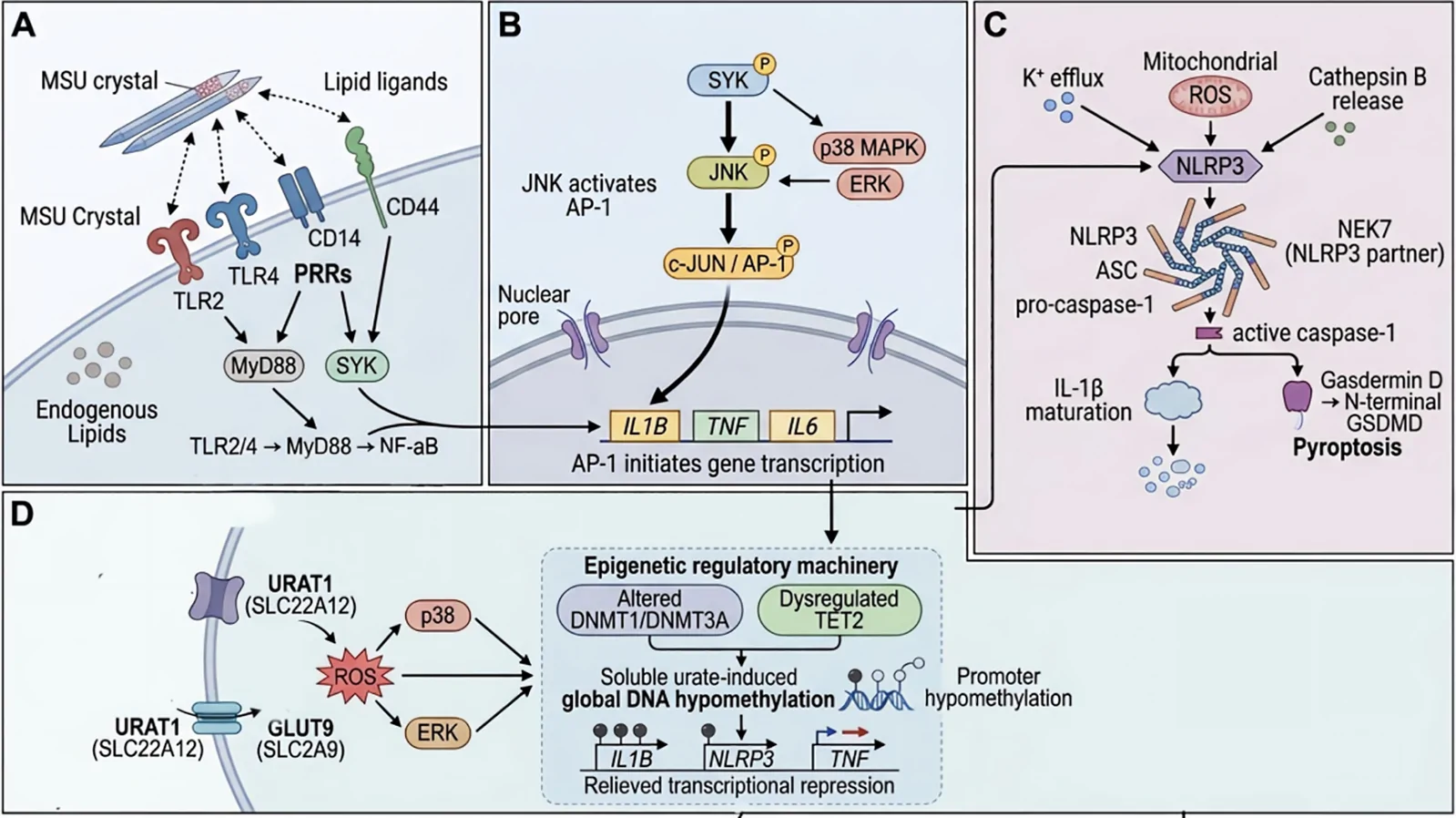
MSU crystals and soluble urate engage distinct yet convergent pathways to establish trained immunity. **(A)** MSU crystals expose specific {011} facets recognized by TLR2, TLR4, CD14, and CD44, triggering both MyD88-dependent (NF-κB priming) and SYK-dependent signalling. **(B)** SYK activates JNK, which phosphorylates c-JUN/AP-1, driving transcriptional reprogramming of pro-inflammatory genes (IL1B, TNF, MMP9, SPP1). **(C)** The NLRP3 inflammasome is assembled upon second signals (K^+^ efflux, mtROS, cathepsin B), leading to caspase-1-mediated maturation of IL-1β and gasdermin D-dependent pyroptosis. **(D)** Soluble urate (without crystallization) enters myeloid cells via URAT1/GLUT9 and induces persistent DNA hypomethylation at promoters of IL1B and NLRP3, lowering the threshold for future activation. Solid arrows indicate mechanisms with direct experimental support; dashed arrows indicate indirect evidence or extrapolation; dotted arrows indicate hypotheses requiring validation in gout.

The mechanistic insights above extend therapeutic implications beyond targeting NLRP3 or IL−1β alone. Modulating the JNK–JUN axis—e.g., via histone deacetylase 3 (HDAC3) deficiency, MCU inhibition, or CD11b activation—shows promise in alleviating MSU−induced inflammation (47). These approaches target the structural sensing and transcriptional reprogramming steps upstream of cytokine release (**Figure 2**), offering potential to disrupt the maladaptive trained state rather than merely suppressing its downstream effector products (47). The distinct features of MSU−driven training versus classical PAMP−driven training are summarized in **Table 2**. Although these findings are based on *in vitro* and murine studies, they provide a plausible mechanistic framework that now requires testing in human gout cohorts.

**Table 2:** Divergent DAMP− and PAMP−triggered innate immune reprogramming via SYK−JNK and mTOR−HIF−1α axes.

Aspect	PAMP-driven trained immunity	MSU crystal-driven trained immunity
Initial trigger	Pathogen-associated molecular pattern (PAMP), typically β-glucan	Damage-associated molecular pattern (DAMP), specifically the {011} crystal face of MSU
Pattern recognition receptor/sensor	Dectin-1 receptor, cooperates with TLR4 signalling	SYK kinase (directly senses crystal geometry)
Core signalling axis	mTOR–HIF-1α axis	SYK–JNK–JUNaxis (TLR4-independent) (15, 45)
Dependence on TLR4/NF-κB	Requires TLR4–NF-κB for transcriptional priming (first signal)	By passesTLR4 and NF-κB; operates autonomously
Metabolic reprogramming	Classical aerobic glycolysis (Warburg effect)	Unique immunometabolic state: upregulation of glycolytic enzymes (HK2, PFKFB3, LDHA) and lysosomal/metabolic gene sets, accompanied by succinate accumulation
Role of NLRP3 inflammasome	Canonical priming signal: NF-κB upregulates NLRP3 and pro-IL-1β	Non-canonical homeostatic function– under controlled conditions supports mitochondrial ETC and limits ROS; when disrupted by MCU-mediated mitochondrial Ca²^+^ overload, shifts to pathological inflammation
Epigenetic reprogramming	HIF-1α-dependent deposition of H3K4me3 at inflammatory gene promoters	JUN binds target promoters, driving a distinct set of inflammatory, lysosomal and metabolic genes
Feedback/self-reinforcement	Relatively linear mTOR–HIF-1α drive	Self-reinforcing circuit: structural recognition → SYK → JNK–JUN → metabolic rewiring + NLRP3-modulated mitochondrial regulation
Therapeutic intervention	Targeting mTOR or HIF-1α (e.g., rapamycin analogues)	Targeting SYK kinase, JNK, MCU, or mitochondrial–inflammasome crosstalk (e.g., MCU inhibitors, HDAC3 modulators)

DAMP, damage−associated molecular pattern; PAMP, pathogen−associated molecular pattern; SYK, spleen tyrosine kinase; JNK, c−Jun N−terminal kinase; mTOR, mechanistic target of rapamycin; HIF−1α, hypoxia−inducible factor 1−alpha; TLR4, Toll−like receptor 4; NF−κB, nuclear factor kappa−light−chain−enhancer of activated B cells; ROS, reactive oxygen species; MCU, mitochondrial calcium uniporter; ETC, electron transport chain; HDAC3, histone deacetylase 3; HK2, hexokinase 2; PFKFB3, 6−phosphofructo−2−kinase/fructose−2,6−biphosphatase 3; LDHA, lactate dehydrogenase A.

### 3.2 The non-inflammatory macrophage paradox

The paradoxical behaviour of non−inflammatory (unpolarized) macrophages towards MSU crystals provides a conceptual foundation for the two−stage model of gout flares, while illuminating the temporal evolution from acute inflammation to chronic trained immunity. We acknowledge that the M1/M2 classification represents an oversimplification of macrophage functional diversity. *In vivo*, macrophage populations exhibit substantial heterogeneity and frequently display mixed or context−dependent phenotypes that do not fit neatly into this binary framework. This is particularly relevant for trained macrophages, which may acquire hybrid functional states that are not adequately captured by the classical polarization paradigm (48). This observation underpins a two−stage model: (i) a priming phase, wherein chronic soluble urate epigenetically reprograms myeloid cells into a “trained but quiescent” state, and (ii) an activation phase, in which a secondary inflammatory challenge (e.g., LPS) unleashes a hyperinflammatory response (49). Notably, MSU crystals alone fail to elicit cytokine secretion from unpolarized macrophages, confirming that crystal deposition is necessary but not sufficient for acute inflammation. In asymptomatic hyperuricaemic individuals, macrophages exhibit enhanced MSU clearance owing to upregulation of lysosomal acidification genes (50). However, co−exposure to LPS and MSU transforms this enhanced phagolysosomal machinery into a driver of amplified IL−1β and TNF−α secretion (50). Thus, hyperuricaemia represents a state of latent immune preparedness; only a subset of patients—those lacking robust anti−inflammatory regulation or harbouring genetic variants that affect JNK–JUN or HDAC3 activity—progress to clinical gout (51).

Early urate−lowering therapy and monitoring of macrophage functional phenotypes may help predict flare risk. Asymptomatic hyperuricaemic individuals exhibit a state of latent immune preparedness: macrophages show enhanced lysosomal acidification and MSU clearance capacity but are epigenetically primed via soluble urate−induced DNA hypomethylation. Monitoring phagocytic index (reflecting crystal clearance capacity) and glycolytic flux (reflecting metabolic activation and trained state) could help stratify patients by flare risk. A high phagocytic index may indicate efficient MSU clearance and lower flare risk, whereas elevated glycolytic flux suggests a trained, hyper−responsive phenotype poised for exaggerated inflammatory responses upon secondary triggers. These functional assays, alongside clinical parameters, could guide early ULT initiation in high−risk asymptomatic hyperuricaemic individuals before epigenetic reprogramming becomes consolidated (19).

The temporal evolution of these reprogramming events can be delineated into three distinct phases. During the acute phase (0–24 h), mitochondrial reactive oxygen species (mtROS) production and NETosis predominate. MSU phagocytosis triggers lysosomal rupture, K^+^ efflux, and mitochondrial dysfunction, culminating in NLRP3 inflammasome activation (52), while neutrophil NETosis releases DAMPs that amplify local inflammation (53). Therapeutic strategies at this stage include mitochondrial antioxidants (e.g., MitoQ) and peptidylarginine deiminase 4(PAD4) inhibitors (54, 55). In the adaptation phase (24–72 h), the metabolic-epigenetic reprogramming detailed above consolidates to sustain the inflammatory state (56). HDAC3 serves as a critical brake, and its deficiency exacerbates inflammation (28, 57, 58). In the chronic phase (>72 h), trained immunity becomes consolidated through stable epigenetic modifications and sustained JNK–JUN activity (59). Mitochondrial Ca²^+^ overload via MCU promotes oxidative stress (52), while haematopoietic stem and progenitor cells (HSPCs) undergo epigenetic reprogramming, continuously generating primed monocytes that perpetuate joint inflammation. Concurrently, endogenous negative regulatory pathways that normally promote tolerance — including upregulation of anti-inflammatory microRNAs (miR-146a, miR-223) and HDAC3-mediated transcriptional repression — become insufficient to counteract the cumulative effect of central and peripheral training. This failure of tolerance consolidation explains why repeated gout flares do not lead to progressive desensitization, but instead often escalate in frequency and severity over time.

Consequently, early intervention is imperative to intercept epigenetic consolidation; delayed treatment may necessitate epigenetic modulators such as HDAC inhibitors or bromodomain and extraterminal domain (BET) bromodomain antagonists (60). Beyond its canonical role in DNA methylation, DNMT3A has recently been implicated in non−methylation−dependent regulation of macrophage inflammatory responses (45) identified a novel AMPK–TFEB/TFE3–DNMT3A/DOT1L axis that regulates lysosomal acidification gene expression in particle−exposed macrophages, revealing that DNMT3A’s function extends to coordinating lysosomal biogenesis and metabolic adaptation independently of its methyltransferase activity. This finding has particular relevance to gout, as it suggests that DNMT3A may modulate the macrophage response to MSU crystals through both methylation−dependent and −independent mechanisms, potentially influencing the balance between crystal clearance and inflammatory activation. The existence of such non−canonical functions further underscores the complexity of epigenetic regulation in gout and opens new avenues for therapeutic intervention that target DNMT3A’s scaffolding or protein−interaction roles rather than its catalytic activity. Systemic metabolic signatures—including lactate, succinate, and acylcarnitines—could serve as biomarkers to guide precision−timed therapy, shifting gout management from reactive symptom control towards proactive immune reprogramming (61).

## 4 Epigenetic reprogramming in gout: molecular evidence from human studies

### 4.1 DNA methylation landscapes in gout

Genome−wide DNA methylation profiling from cross−sectional clinical studies reveals widespread differential methylation in immune cells of gout patients compared with healthy controls, defining a distinct ‘gout methylome’ enriched in immune−related pathways including Th17 cell differentiation and osteoclastogenesis (16, 27). These methylation changes are of particular interest because they represent a more stable epigenetic layer than dynamic histone marks, potentially serving as a durable molecular record of prior urate exposure. Notably, DNA methylation at CpG islands can persist through cell division, providing a mechanism for maintaining trained phenotypes across generations of myeloid cells. These findings indicate a close association between DNA methylation reprogramming and gout pathogenesis.

However, it remains challenging to establish causal direction from current human data: most available studies are cross−sectional and cannot confirm whether epigenetic alterations precede disease onset, or if they are secondary consequences of chronic inflammation and hyperuricemia (62). Prospective longitudinal cohorts tracking high−risk hyperuricemic individuals are still needed to clarify the temporal sequence of DNA methylation changes and gout development (63).These cross−sectional methylation findings represent epigenetic associations rather than demonstration of bona fide trained immunity. They show that urate exposure correlates with persistent epigenetic changes, but they do not by themselves establish that these changes confer a functionally altered response upon rechallenge after a stimulus−free interval.

Preclinical interventional studies provide preliminary support for a causal role. Broad−spectrum DNA methyltransferase inhibitors (DNMTi) have been shown to reverse urate−induced myeloid hyper−responsiveness in both *in vitro* cell culture systems and *in vivo* murine gout models. These proof−of−concept findings demonstrate that DNA methylation changes can functionally modulate inflammatory responses, but further clinical validation is required to confirm their causal contribution to human gout (16).

The reversibility of this phenotype highlights the dynamic nature of the epigenome and opens a promising therapeutic avenue: the potential to reset the maladaptively trained immune system in gout patients through targeted epigenetic modulation (64). Such an approach moves beyond symptom management or urate lowering and aims to correct the underlying immune dysregulation that defines the disease’s chronicity (16, 65).

### 4.2 Histone modification signatures

The investigation of histone modification signatures in gout has unveiled a critical layer of epigenetic regulation that governs the inflammatory response to monosodium urate (MSU) crystals, positioning class I histone deacetylases (HDACs)—particularly HDAC3—as central molecular switches in disease pathogenesis. The core mechanism involves the dynamic acetylation and deacetylation of histone tails, which directly controls chromatin accessibility and the transcriptional activity of pro-inflammatory genes. In the context of gout, MSU crystals act as potent danger signals that trigger a cascade of intracellular events culminating in the activation of the NLRP3 inflammasome and NF-κB signalling. Histone modifications serve as a crucial regulatory checkpoint for these pathways.

Pharmacological inhibition of class I HDACs significantly dampens MSU crystal−induced inflammation through accumulation of H3K9/K14ac at promoters and enhancers of Il1b, Il6, and Tnfa (66). HDAC3, a class I member, exhibits dichotomous, gene−specific functions—its catalytic activity depends on NCoR1/2 corepressors, and it both represses and activates distinct target genes to fine−tune inflammatory responses (67).

In a murine gout model, macrophage−specific HDAC3 knockout alleviates MSU−induced inflammation by suppressing NLRP3 activation and reducing neutrophil recruitment (66). *In vitro* validation in human primary monocytes and macrophages further supports this regulatory role of HDAC3 in inflammatory responses (68). Collectively, these preclinical findings support a model in which HDAC3 integrates MSU signals and shapes the epigenetic landscape at pro−inflammatory loci, potentially orchestrating a maladaptive trained immunity program in myeloid cells that primes hyper−responsiveness to recurrent challenges.

This central role makes HDAC3 and related class I HDACs highly attractive therapeutic targets. Unlike broad-spectrum anti-inflammatory drugs that can have significant off-target effects, selective HDAC3 inhibitors offer the potential for a more precise intervention that disrupts the core epigenetic machinery driving gouty inflammation without completely shutting down essential immune functions. The success of HDAC3 KO in murine models provides strong preclinical proof-of-concept for this strategy (66). By targeting the epigenetic root of the inflammatory cascade, such an approach could not only treat acute flares but also modify the long-term course of the disease by preventing the establishment of pathological immune memory—a paradigm shift from merely managing symptoms to addressing the fundamental dysregulation of the innate immune system that characterizes chronic gout (22).

### 4.3 Single-cell and spatial transcriptomics: the TGAMs discovery

Integrating single−cell RNA−sequencing and spatial transcriptomics on 44,221 synovial cells from gout patients, the study identified a novel macrophage subset—tophaceous gout−associated macrophages (TGAMs)—defined by expression of SPP1, MMP9, and CHI3L1 (69). TGAMs localize specifically to the corona zone of tophi, the interface between MSU crystals and host immune cells, positioning them to sense crystal−derived signals and orchestrate tissue remodelling (69, 70). Their pro−inflammatory and ECM−degrading phenotype represents a stable, epigenetically reprogrammed trained−like state that persists even after crystal clearance, perpetuating chronic inflammation and joint damage (71, 72). This discovery provides a concrete cellular substrate for the global epigenetic alterations in gout and directly links trained immunity theory to human pathology. Therapeutically, TGAMs’ unique surface markers offer opportunities for selective depletion or modulation, potentially disrupting the self−sustaining inflammatory loop without broad immunosuppression (69).

### 4.4 Innate-adaptive crosstalk in gout pathogenesis

The pathogenesis of gout extends beyond MSU crystal deposition; it is driven by a maladaptive dialogue between innate and adaptive immunity. While the initial trigger—MSU crystals—activates macrophages via NLRP3 and IL−1β release, the chronicity and systemic manifestations are amplified through crosstalk with adaptive immunity, with Th17 cells playing a pivotal role (69).

trained immunity in gout endows myeloid cells with a hyper−responsive phenotype, producing IL−1β, IL−6, and TNF−α. These cytokines create a local milieu conducive to Th17 differentiation from naïve CD4+ T cell precursors, with IL−1β and IL−6 (in concert with TGF−β) driving the transcriptional program that commits T cells to the Th17 lineage (73).

The central role of Th17 cells is established through studies on HIF−1α. In CD4+ T cells, HIF−1α acts as a metabolic switch, driving glycolysis—a pathway essential for effector functions. HIF−1α directly promotes Th17 differentiation while suppressing alternative fates such as regulatory T cells (Tregs) (74). Genetic ablation of HIF−1α specifically in CD4+ T cells profoundly alleviates gout symptoms in experimental models, reducing serum uric acid, diminishing Th17 polarization, and mitigating renal injury (74). The reduction in uric acid suggests a feedback loop whereby adaptive immune activation influences the core metabolic defect of hyperuricemia, and the protection from renal injury underscores the systemic nature of gout’s immune dysregulation.

This intricate interplay creates a self-reinforcing inflammatory circuit. Innate immune cells, trained by urate, produce cytokines that drive Th17 expansion. In turn, Th17 cells secrete IL-17, which acts on stromal cells, synoviocytes, and other immune cells to induce the production of additional pro-inflammatory mediators (e.g., IL-6, G-CSF, chemokines) and matrix-degrading enzymes, thereby amplifying tissue inflammation, neutrophil recruitment, and bone erosion (75).

The discovery of this innate−adaptive axis, centred on HIF−1α−driven Th17 responses, provides a unifying framework that integrates the initial innate danger−sensing event with the long−term adaptive immune memory and tissue remodelling processes that define advanced gout. It highlights that effective therapeutic strategies may need to target not only the innate triggers (e.g., via HDAC3 inhibition) or the core metabolic defect (urate−lowering therapy) but also this critical adaptive amplifier to fully disrupt the pathogenic cascade (41).

### 4.5 Spatial and temporal immune gradients

The spatiotemporal immune framework reconciles the acute, episodic nature of gout flares with underlying chronicity and systemic manifestations, with trained immunity serving as the mechanistic link between temporal and spatial dimensions (22).

In the temporal domain, the gout flare follows a defined sequence. Acute phase: resident macrophages recognize MSU crystals, triggering NLRP3 activation and IL-1β secretion, which recruits neutrophils and drives classic inflammation. Resolution phase: active programs, alternative activation states, aggNET formation, and specialized pro-resolving mediators (SPMs), restore tissue homeostasis (76). Chronic intercritical phase: clinical remission does not signify return to baseline; persistent low-grade activation of circulating monocytes and tissue-resident macrophages — hallmarks of trained immunity — maintains a hyper-responsive phenotype primed for future flares (64).

This temporal model is linked to a spatial gradient of immune activation. At the epicentre (the joint), inflammation is focal and intense. In adjacent bone marrow and peri−articular tissues, the environment is less acutely inflamed but harbours trained myeloid precursors. In the systemic circulation, monocytes from gout patients in remission display epigenetic and functional signatures of trained immunity, including altered histone modifications and heightened cytokine production capacity (77).

**Figure 3:**
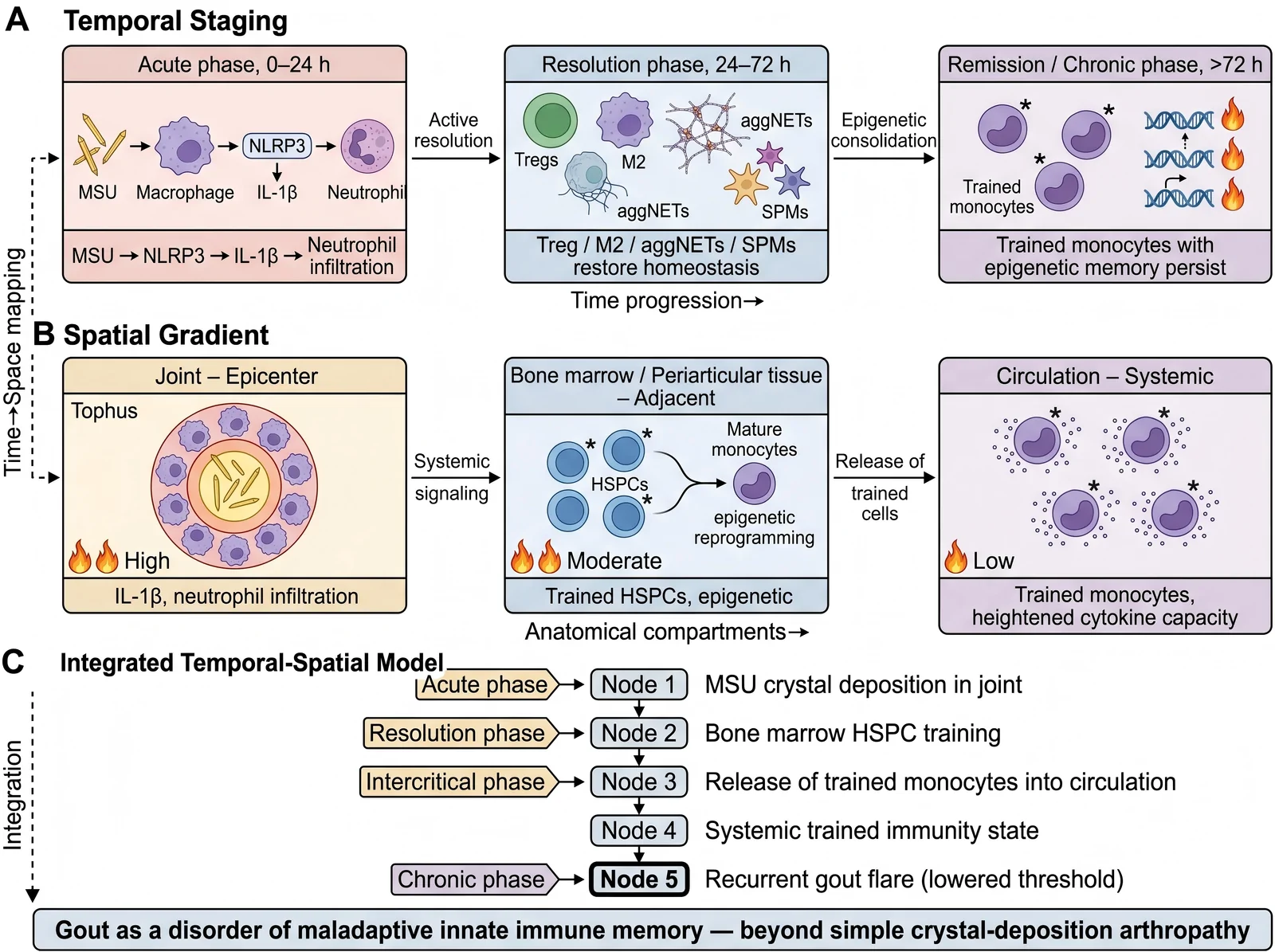
Spatiotemporal immune framework of gout. **(A)** Temporal phases: acute, resolution, and intercritical/chronic; **(B)** Spatial gradient; **(C)** Spatiotemporal integration. Solid arrows indicate mechanisms with direct experimental support; dashed arrows indicate indirect evidence or extrapolation; dotted arrows indicate hypotheses requiring validation in gout.

Thus, local MSU−driven inflammation acts as a training ground that systemically reprograms the myeloid lineage via cytokine signalling (IL−1β, GM−CSF) to the bone marrow, promoting release of epigenetically primed cells into circulation. These trained cells serve as a reservoir of hyper−responsive effectors, lowering the threshold for new flares upon minor crystal deposition or secondary triggers—a mechanism explaining the chronic, relapsing nature of gout (**Figure 3**) (16).

## 5 From local joints to systemic inflammation: trained immunity as a unifying mechanism for gout comorbidities

Gout extends beyond the joints, with systemic inflammation linking it to a wide spectrum of comorbidities. trained immunity provides a unifying framework explaining how a localized articular disease evolves into a systemic inflammatory state with multisystem consequences (78).

### 5.1 The cardiovascular paradox

Gout patients face substantially elevated cardiovascular (CV) risk, with hyperuricemia serving as an independent risk factor for CVD and mortality (79). This elevated risk arises from multiple interconnected mechanisms. First, soluble urate directly impairs endothelial function by reducing NO bioavailability and inducing oxidative stress, promoting vasoconstriction and hypertension. Second, urate−driven NLRP3 inflammasome activation and TLR signalling produce pro−inflammatory cytokines (IL−1β, TNF−α, IL−6) that are also key drivers of atherosclerosis, promoting endothelial activation, monocyte recruitment, foam cell formation, and plaque instability. Third, systemic trained immunity generates hyper−responsive monocytes that infiltrate atherosclerotic plaques and sustain vascular inflammation even in the absence of local coronary MSU deposition. Fourth, gout shares common comorbidities—obesity, metabolic syndrome, hypertension, diabetes, and CKD—that independently drive atherosclerosis. The failure of ULT alone to reduce CV risk underscores that inflammation−driven mechanisms, rather than urate itself, are likely the dominant drivers of cardiovascular disease in gout (79). Conditions predisposing to gout—obesity, metabolic syndrome, diabetes, hypertension, and CKD—also drive atherosclerosis (80). Despite this association, urate-lowering therapies (ULT) have not convincingly improved CV outcomes; recent meta-analyses show equivocal CV benefit even when renal outcomes improve (79, 81). This shifts attention from urate itself toward inflammation-driven mechanisms connecting gout to atherosclerosis (82).

Another line of evidence challenges the simplistic notion that MSU crystals deposited directly in coronary arteries drive CVD. There is a lack of convincing data for clinically significant monosodium urate crystal (MSU crystals) deposition in atherosclerotic plaques (83). This suggests that alternative mechanisms—beyond local crystal deposition—must account for the heightened cardiovascular risk observed in gout.

Mechanistic insights explain persistent CV risk even with controlled urate. First, vascular endothelial dysfunction emerges early in intercritical gout; patients exhibit proinflammatory transcriptional profiles in endothelial cells, diminished flow−mediated dilation, and elevated hs−CRP (84, 85). Neutrophils from these patients show activation signatures mirroring trained immunity, reinforcing persistent innate immune activation (86). Second, MSU crystal deposition can occur in coronary arteries as well as joints, as revealed by recent advanced imaging techniques. While such deposits may not directly form atheromatous plaques, they create a proinflammatory vascular niche that accelerates plaque progression through paracrine mechanisms and sustained cytokine release (87). Third, elevated levels of proinflammatory cytokines—IL-1β, TNF-α, and IL-6—that are central to gout pathophysiology are also key drivers of atherosclerosis at multiple stages. This shared inflammatory machinery underscores the need to understand how joint-specific MSU crystals-triggered inflammation expands systemically (88). The proposed joint–bone marrow–vascular axis integrates current evidence but includes several steps that remain hypothetical in the gout context and require direct experimental validation.

The failure of ULT alone to resolve CV risk redirects attention to the inflammatory cascade. A mechanistic framework posits that expansion of local articular to systemic inflammation—driven by MSU crystals−activated macrophages—accelerates atherosclerosis. Trained monocytes infiltrate plaques and sustain vascular inflammation, making NLRP3 products and trained monocytes plausible therapeutic targets for simultaneously addressing gout flares and CV risk (82, 89).

### 5.2 Clonal haematopoiesis (CHIP): the aging link

Aging−related somatic mutations in clonal haematopoiesis of indeterminate potential (CHIP)—particularly in DNMT3A and TET2—are among the most exciting recent discoveries in gout research (90). CHIP, defined by somatic mutations in leukaemia driver genes at VAF ≥2% without overt blood disorder, is increasingly recognized as a systemic, age−associated risk factor for non−malignant diseases (91). CHIP−associated mutations alter epigenetic programs, skew myelopoiesis, and increase proinflammatory cytokines, driving chronic inflammation and immune imbalance (92).

The link between CHIP and inflammation is well established. Age-related DNMT3A and TET2 mutations accumulate in hematopoietic stem cells (HSCs). These mutations confer epigenetic and metabolic advantages, skewing haematopoiesis toward a pro-inflammatory myeloid lineage and driving persistent inflammation (93). These myeloid derivatives produce increased IL-1β and IL-6, establishing a pro-atherogenic environment, contributing to plaque instability, and accelerating vascular aging. In the context of gout, CHIP mutations disrupt the epigenetic restraint of MSU crystals-induced myeloid inflammation, thereby accelerating atherosclerosis (82).

Epidemiological data from large biobanks substantiate this mechanistic framework. Analysis of the MGB Biobank and UK Biobank demonstrates that individuals with CHIP have a significantly higher frequency of gout (odds ratio 1.69 for VAF≥2%; hazard ratio 1.28 for incident gout in UKB (90). However, as Merriman and Joosten have cautiously noted, while CHIP mutations provide a compelling mechanistic link, direct demonstration of trained immunity in gout patients remains to be established. The association does not by itself prove that CHIP drives a trained phenotype; it is equally compatible with other inflammation−amplifying mechanisms. Mechanistic insights from murine models and *in vitro* assays further support a causal model: age−related accumulation of CHIP mutations may progressively dismantle epigenetic restraint on MSU−induced myeloid inflammation, thereby amplifying gouty inflammation and its cardiovascular comorbidities (90). Similarly, Tet2-knockout macrophages elaborate significantly higher IL-1β levels in response to MSU crystals *in vitro*, an effect that is ameliorated by both genetic and pharmacologic NLRP3 inhibition (90). Single-cell multiomics analyses have further revealed that DNMT3A- and TET2-mutant clonal haematopoiesis exert divergent effects on inflammatory responses, with TET2 deficiency particularly amplifying NLRP3-dependent responses (94). Nevertheless, these interpretations remain inferential, and direct evidence for CHIP−mediated trained immunity in gout patients is still awaited.

**Figure 4:**
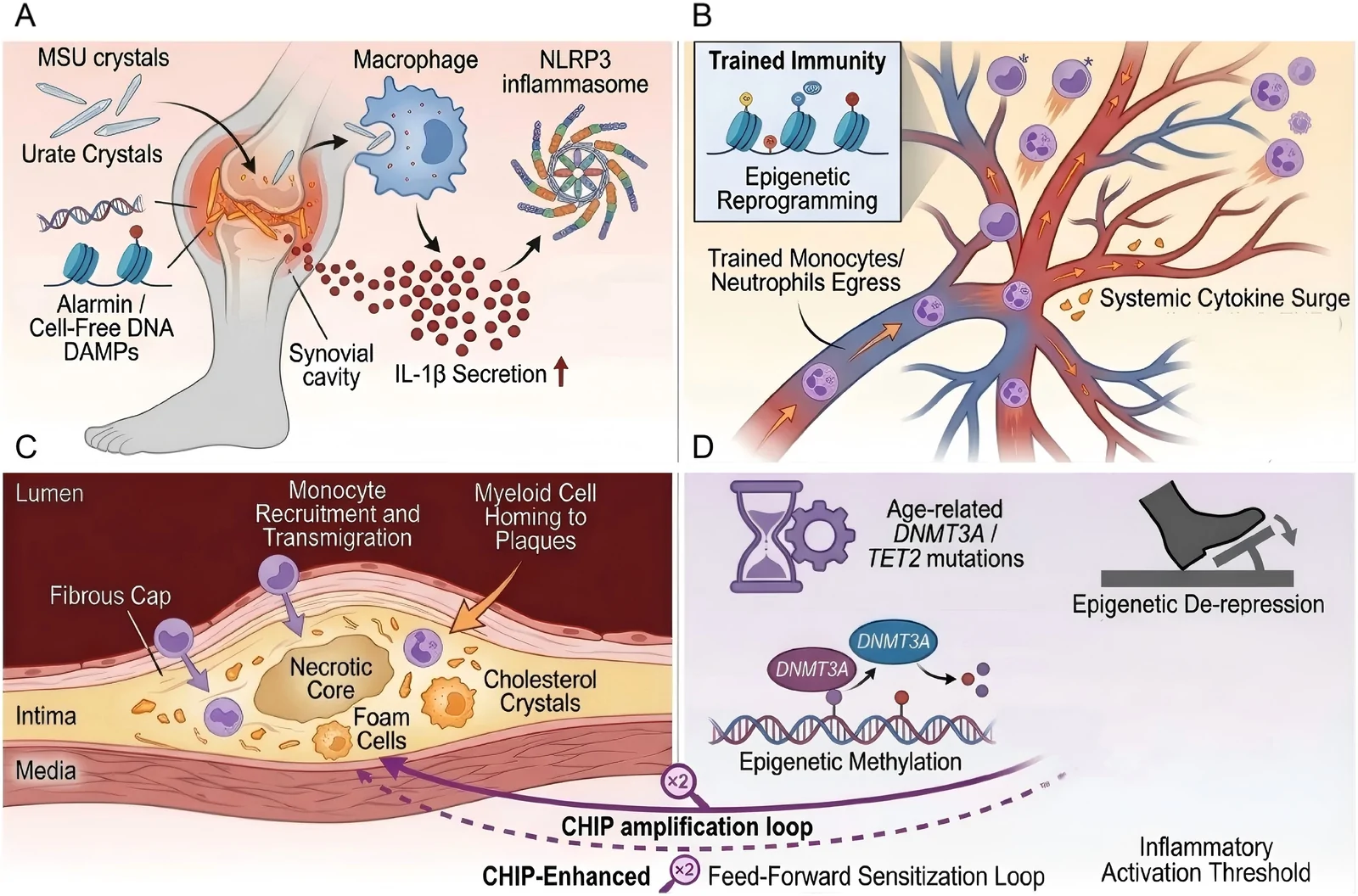
Visual model of systemic inflammation coupling gout to atherosclerosis. **(A)** MSU crystal deposition and macrophage training in joints; **(B)** Release of trained monocytes into circulation; **(C)** Infiltration of trained myeloid cells into the vascular wall (lumen–intima–media), driving atherosclerotic plaque formation and instability; **(D)** Epigenetic ‘brake’ failure (e.g., CHIP mutations, HDAC3 deficiency) and therapeutic reprogramming, which directly modulates the vascular inflammatory response shown in **(C)**.

Epidemiological data from large human biobanks consistently shows an association between CHIP (predominantly DNMT3A and TET2 mutations) and increased gout risk. Mechanistic insights from murine models and *in vitro* assays further support a causal model: age−related accumulation of CHIP mutations may progressively dismantle epigenetic restraint on MSU−induced myeloid inflammation, thereby amplifying gouty inflammation and its cardiovascular comorbidities (82). The CHIP pathway is involved in control of the epigenome—DNA methylation, histone modification, and metabolism of substrates—and may make people more susceptible to gout by causing the innate immune system to be hyper-responsive to MSU crystals. This explains why aging increases both gout and cardiovascular risk, and why older gout patients—who are more likely to harbour CHIP mutations—remain at elevated cardiovascular risk even after serum urate levels are normalized (82, 90). These findings identify CHIP as a critical amplifier of NLRP3-dependent inflammatory responses to MSU crystals in patients with gout (**Figure 4**) (64).

### 5.3 Other extra-articular manifestations

#### 5.3.1 Gout-associated nephropathy

The relationship between gout and chronic kidney disease (CKD) is bidirectional and increasingly recognized as a critical clinical challenge (95). Hyperuricemia is an independent risk factor for CKD, and emerging evidence highlights dysfunctional macrophages as a potential bridge between hyperuricemia and renal injury. Macrophages contribute to hyperuricemia-induced complications through three primary mechanisms: direct action on urate transporters such as URAT1 and ABCG2, modulation of inflammation through TLRs and the NLRP3 inflammasome, and effects on oxidative stress mediators (96).

The pathophysiology of urate−induced kidney injury extends beyond crystal deposition in the renal interstitium and collecting ducts. Soluble urate itself directly injures renal tubular cells by inducing oxidative stress, mitochondrial dysfunction, and apoptosis, and by activating intrarenal NLRP3 inflammasomes even in the absence of frank crystallisation. Urate impairs renal microvascular function through endothelial dysfunction and afferent arteriolar vasoconstriction, reducing renal blood flow and promoting ischaemic injury. Systemic trained immunity further amplifies renal injury, as trained myeloid cells produce IL−1β and other cytokines that circulate and drive tubulointerstitial inflammation and fibrosis. Additionally, emerging evidence implicates gasdermin−mediated pyroptosis in crystalline nephropathy, linking the same cell death pathway activated by MSU crystals in joints to renal pathology. Chronic low-grade systemic inflammation driven by trained myeloid cells exacerbates renal fibrosis through sustained cytokine production. Elevated uric acid activates intrarenal NLRP3 inflammasomes, promoting tubular injury and interstitial inflammation even in the absence of frank crystal deposition. Moreover, emerging evidence implicates gasdermin-mediated pyroptosis in crystalline nephropathy, linking the same cell death pathway activated by MSU crystals in joints to renal pathology (97).

The clinical management of hyperuricemia in CKD is complicated by the inconsistent results of randomized controlled trials (95). Some studies have not confirmed the benefits of ULT in slowing CKD progression, leading to cautious recommendations in clinical guidelines (98, 99). However, a 2025 network meta-analysis found that allopurinol and febuxostat are effective in reducing composite renal events and improving renal function in asymptomatic hyperuricemia (41). These contrasting findings highlight the heterogeneity of CKD populations and the need for phenotype-guided treatment strategies (100). Recent advances in understanding the pathophysiology of hyperuricemia in CKD—including the recognition that CKD-associated hyperuricemia may arise from heterogeneous excretion defects such as insufficient glomerular filtration and excessive tubular reabsorption—are beginning to inform more individualized approaches to urate-lowering therapy (100). Trained immunity may further contribute to this heterogeneity: patients with evidence of myeloid reprogramming may derive greater benefit from early ULT or adjunctive anti-inflammatory therapy.

#### 5.3.2 Metabolic syndrome exacerbation

Gout is closely associated with metabolic syndrome (MetS)—central obesity, insulin resistance, hypertension, and dyslipidaemia—through a bidirectional pathogenic loop (101). Elevated uric acid impairs insulin sensitivity and β−cell function via oxidative stress and inflammation, while insulin resistance reduces renal urate excretion, forming a vicious metabolic cycle (49, 101). At the molecular level, hyperuricemia inhibits IRS1 and Akt phosphorylation, reducing peripheral glucose uptake, while also promoting endothelial dysfunction and adipocyte dysregulation (102).

**Figure 5:**
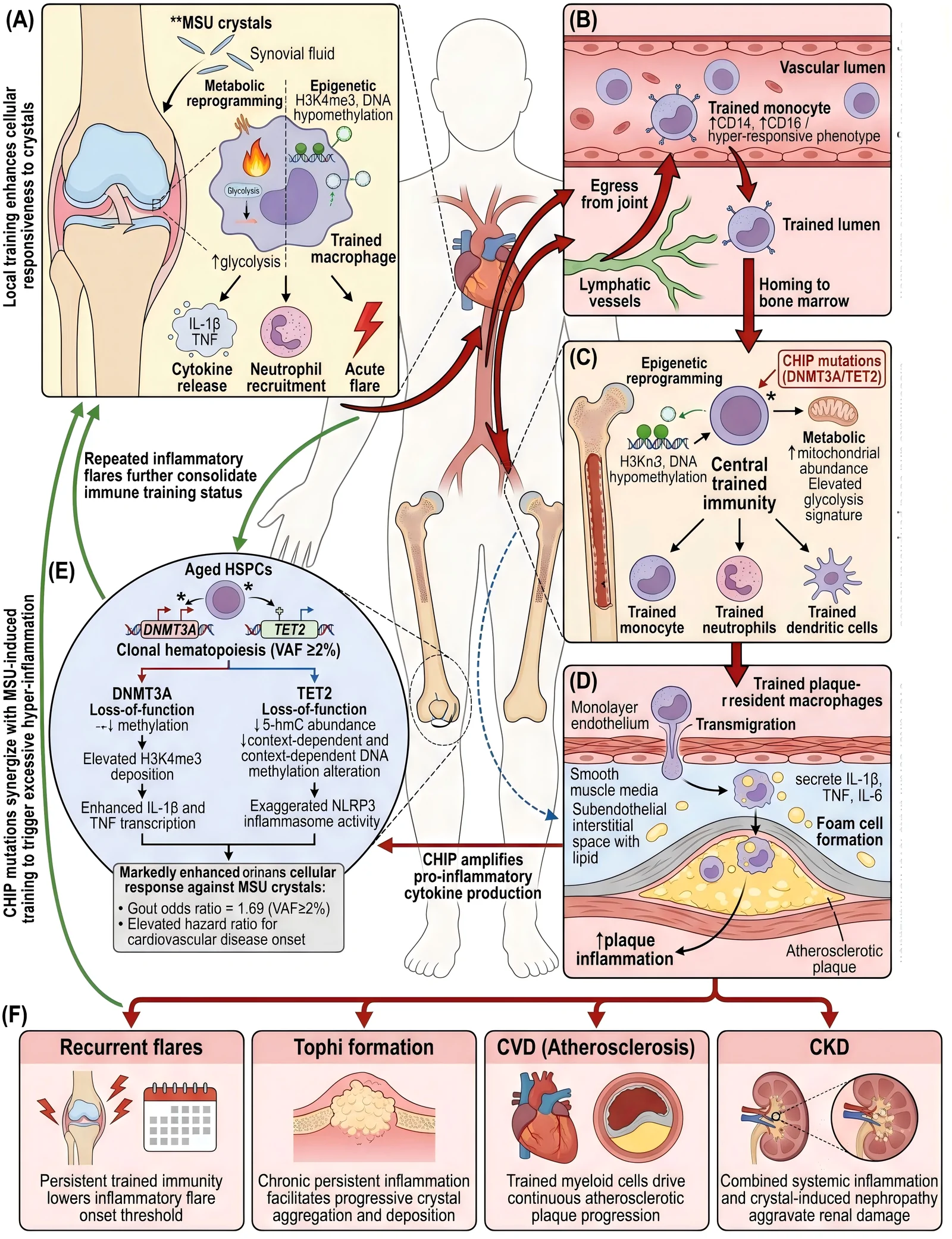
A comprehensive illustration of the progression from local articular training to systemic inflammation and atherosclerosis. **(A)** joint-level MSU crystal deposition and macrophage training; **(B)** release of trained monocytes into circulation; **(C)** bone marrow training of HSPCs; **(D)** infiltration of trained myeloid cells into atherosclerotic plaques; **(E)** CHIP mutations (DNMT3A/TET2) as amplifiers; **(F)** clinical outcomes: recurrent flares, tophi, CVD, CKD. Solid arrows indicate mechanisms with direct experimental support; dashed arrows indicate indirect evidence or extrapolation; dotted arrows indicate hypotheses requiring validation in gout.

Gut microbiota dysregulation emerges as a unifying link. Multi−omics integration reveals distinct microbiota–metabolite profiles in gout patients with concurrent MetS, suggesting the microbiota–bile acid–inflammation axis bridges these conditions (103–106). trained immunity amplifies this crosstalk: trained macrophages within adipose tissue promote chronic low−grade inflammation, perpetuating insulin resistance and further impairing urate excretion (107, 108). This creates a feed−forward loop—hyperuricemia−induced inflammation trains myeloid cells, which worsen metabolic parameters and raise urate levels through reduced excretion and increased purine turnover as depicted in **Figure 5**. Thus, targeting trained myeloid cells—rather than simply lowering urate—could simultaneously address gouty inflammation and its metabolic comorbidities (59).

#### 5.3.3 Neuroinflammatory complications

While the evidence remains more speculative, trained immunity may also contribute to neuroinflammatory consequences in gout. Systemic inflammation driven by trained myeloid cells can activate microglia—the resident macrophages of the central nervous system—through the release of circulating cytokines and disruption of the blood-brain barrier. Although clinical data directly linking gout to neurodegenerative diseases are sparse, gout shares with other chronic inflammatory conditions the potential for sustained systemic inflammation to influence central nervous system function. Recent reviews have extended the discussion of CHIP to include neurodegenerative conditions, with epigenetic dysregulation and trained immunity emerging as potential common denominators across multiple inflammation-driven diseases. This represents an important area for future investigation, particularly as the population ages and the cumulative burden of chronic inflammation on the aging brain becomes increasingly recognized (109).

## 6 Therapeutic translation: targeting trained immunity in gout

trained immunity is evolutionarily conserved for host defence (108), but in gout it becomes maladaptive—MSU crystals and soluble urate persistently rewire myeloid epigenetics and metabolism, sustaining joint inflammation and systemic comorbidities (107, 108). Complete ablation of innate immune function is undesirable; the therapeutic goal must instead be to reprogramme trained cells back towards homeostasis, restoring normal responsiveness while preserving protective immunity (59, 108). From a clinical perspective, interventions targeting trained immunity in gout span two tiers: conventional urate-lowering therapy that removes the upstream training trigger, and mechanism-driven targeted strategies that directly modulate epigenetic, metabolic and inflammatory nodes of innate immune memory.

The anti−inflammatory mechanisms of colchicine in gout may extend beyond canonical NLRP3–IL−1β inhibition. Using primary human peripheral blood mononuclear cells (PBMCs) and monocytes stimulated with MSU crystals, Merlo Pich et al. recently demonstrated that colchicine reduces the secretion of several chemokines, notably MCP−1, while having no inhibitory effect on the release of proinflammatory cytokines such as IL−1β, IL−6, or TNF (110). Both MCP−1 messenger RNA expression and intracellular protein concentrations were inhibited by colchicine, suggesting an effect at the transcriptional level. Lower chemokine production was responsible for reduced chemoattraction of monocytes. Moreover, colchicine attenuated ROS production and phagocytic activity in monocytes, alongside suppressing glycolytic metabolism and oxidative phosphorylation (110). These findings suggest that some clinically effective therapies may exert their beneficial effects through pathways that are not necessarily dependent on IL−1β, broadening the therapeutic target landscape beyond the inflammasome.

### 6.1 Conventional urate-lowering therapies: stage-dependent modulation of trained immunity

As the cornerstone of chronic gout management, urate-lowering therapies (ULT) — including xanthine oxidase inhibitors (allopurinol, febuxostat) and uricosuric agents (benzbromarone) — exert indirect effects on trained immunity by eliminating the upstream trigger: soluble urate and MSU crystals. However, their capacity to reverse established innate immune memory is heterogeneous and highly dependent on disease stage and the degree of epigenetic consolidation (16, 64).

In early-stage hyperuricemia and newly diagnosed gout, sustained urate lowering can partially reverse soluble urate-induced DNA hypomethylation in circulating monocytes, according to small clinical observational studies. Parallel ex vivo functional assays show reduced cytokine hyper-responsiveness to TLR stimulation after 6–12 months of effective ULT, suggesting that removal of the persistent trigger can gradually attenuate peripheral trained immunity, especially when epigenetic reprogramming has not been stably consolidated (16, 49). Allopurinol, for example, has been associated with reduced H3K4me3 enrichment at pro-inflammatory gene promoters in monocytes from treatment-naive gout patients, alongside decreased flare frequency — an effect consistent with its ability to dampen glycolytic flux and succinate accumulation, two upstream drivers of activating histone marks (25, 111).

In contrast, ULT alone rarely fully erases the trained immune phenotype in patients with long-standing tophaceous gout, even after years of sustained serum urate normalization. This aligns with the central training model: once durable epigenetic reprogramming is established at the HSPC level in bone marrow, elimination of peripheral urate triggers cannot readily reset the myeloid differentiation bias. Residual trained tissue-resident macrophages and continuously generated primed monocytes maintain a hyper-responsive state, providing a mechanistic explanation for breakthrough flares despite optimal serum urate control (13, 14).

Notably, preliminary preclinical data suggest that certain ULT agents may have direct off-target immunomodulatory effects independent of urate lowering. For instance, febuxostat has been reported to exert off−target immunomodulatory effects independent of urate lowering. *In vitro* studies indicate that febuxostat inhibits NLRP3 inflammasome assembly by reducing intracellular ROS production, a critical trigger for inflammasome activation. Additionally, febuxostat may modulate the mTOR/HIF−1α axis, thereby suppressing glycolytic reprogramming in macrophages and potentially dampening the metabolic foundation of trained immunity (24, 42). However, these observations are derived from *in vitro* experiments and murine models; whether febuxostat meaningfully modulates trained immunity in gout patients remains unconfirmed and requires dedicated clinical investigation (24, 42). However, whether this translates to meaningful modulation of trained immunity in humans remains unconfirmed. Overall, clinical evidence directly linking ULT to epigenetic reversal of trained immunity is limited to small observational studies; large prospective trials with dedicated epigenetic and functional endpoints are required to quantify the magnitude and durability of this effect (65, 111).

### 6.2 Targeted therapeutic strategies against trained immunity

Beyond upstream urate reduction, multiple mechanism-driven strategies directly target the core machinery of trained immunity. They fall into five interconnected intervention domains: (i) epigenetic modifications, (ii) metabolic rewiring, (iii) myeloid differentiation bias, (iv) inflammatory cytokine output, and (v) persistence of immune memory (59, 108). Representative agents, mechanisms and limitations are summarized in **Table 3**.

**Table 3:** Overview of therapeutic strategies targeting trained immunity in gout.

Strategy/Target class	Representative agents	Primary mechanism	Major limitations/risks
IL-1β blockade	Canakinumab, Anakinra, Rilonacept	Neutralise IL-1β or block its receptor	Does not erase trained memory; relapse upon cessation; no effect on subclinical systemic inflammation
Metabolic modulators	Metformin, Dioscin, Statins	AMPK/mTOR inhibition (metformin); HIF-1α suppression (dioscin); mevalonate pathway inhibition (statins)	GI intolerance (metformin); limited human data (dioscin); no dedicated RCTs for gout outcomes (statins)
Epigenetic reprogrammers	HDAC inhibitors (TSA, butyrate); DNMT inhibitors (5-azacytidine); BET inhibitors (JQ1)	Reverse H3K4me3/H3K27ac or DNA methylation; block acetyl-lysine reading	Systemic toxicity (thrombocytopenia, GI, cardiac); genotoxic risks (DNMTi); CHIP mutations may paradoxically expand mutant clones
NLRP3/downstream effectors	MCC950 (discontinued); NEK7 degraders; PAD4 inhibitors (GSK-484)	Block inflammasome assembly (NLRP3/NEK7); inhibit NETosis (PAD4)	MCC950: hepatotoxicity; NEK7 degraders still preclinical; PAD4i effects on infection risk unknown
Emerging & holistic	Uricase + tolerance induction; Probiotics (butyrate producers); Exosome inhibitors (GW4869)	Antigen-specific immune tolerance; gut–joint axis (miR-146a/HDAC); block trained-phenotype transfer	Tolerance regimen complex; probiotics need larger RCTs; exosome targeting lacks specificity
Colchicine	Colchicine	Inhibits chemokine production (notably MCP−1) via transcriptional suppression; attenuates ROS production, phagocytic activity, and glycolytic metabolism in monocytes; effects are largely independent of IL−1β/IL−6/TNF inhibition (110)	Narrow therapeutic window; GI intolerance; drug interactions; does not directly erase trained epigenetic memory

Colchicine, while commonly used for gout flares, exerts anti−inflammatory effects that are largely independent of IL−1β inhibition. Recent evidence shows that colchicine reduces chemokine production (notably MCP−1) and attenuates ROS production, phagocytosis, and glycolytic metabolism in MSU−stimulated monocytes without inhibiting IL−1β, IL−6, or TNF release (110). IL−1β, interleukin−1 beta; AMPK, AMP−activated protein kinase; mTOR, mechanistic target of rapamycin; HIF−1α, hypoxia−inducible factor 1−alpha; RCTs, randomized controlled trials; HDAC, histone deacetylase; DNMT, DNA methyltransferase; BET, bromodomain and extraterminal domain; GI, gastrointestinal; CHIP, clonal haematopoiesis of indeterminate potential; DNMTi, DNA methyltransferase inhibitors; NLRP3, NOD−, LRR− and pyrin domain−containing protein 3; NEK7, NIMA−related kinase 7; PAD4, peptidylarginine deiminase 4; miR, microRNA.

**Figure 6:**
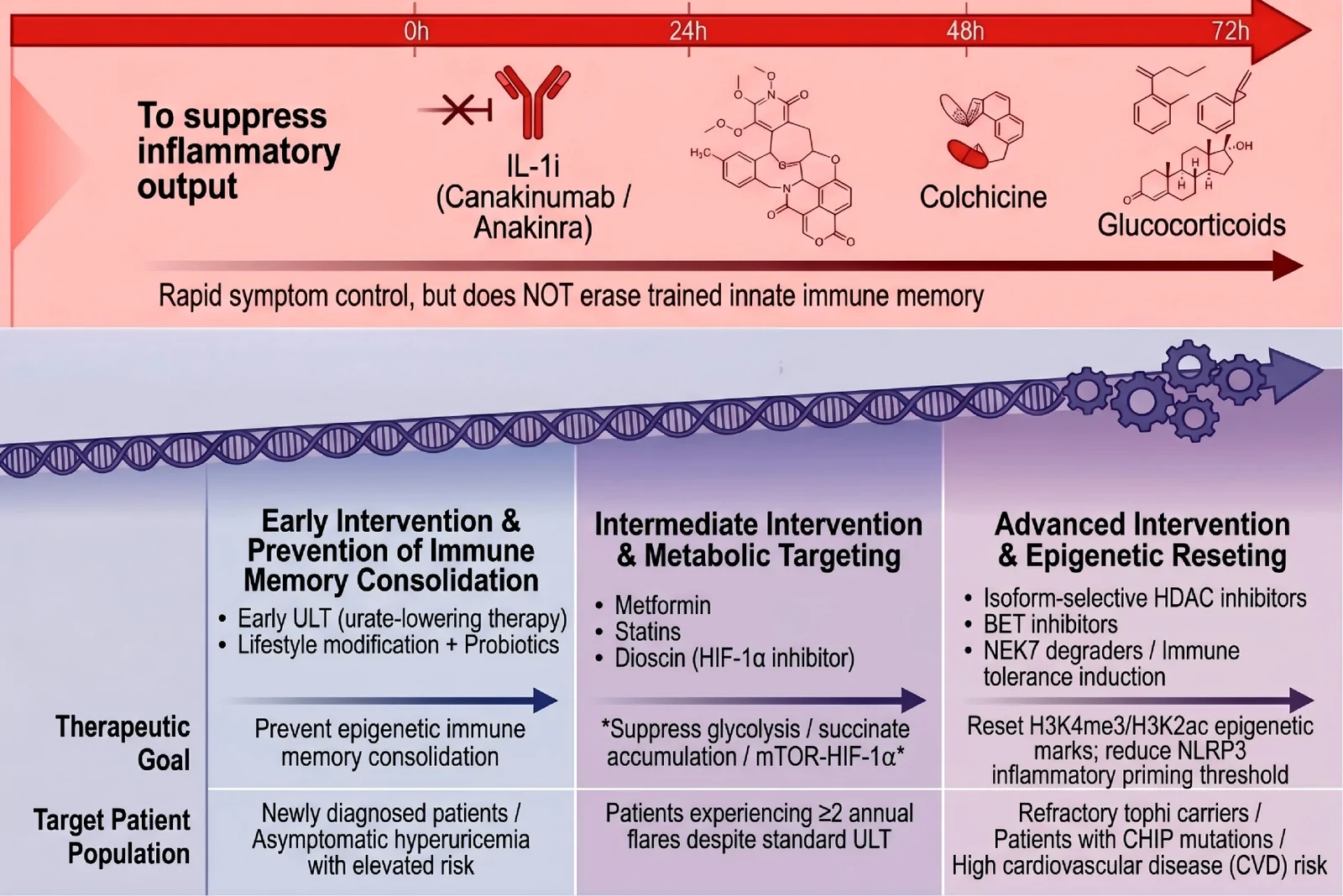
Dual−track strategy for targeting trained immunity in gout.

Despite these diverse options, several challenges remain. First, no validated biomarkers currently quantify the trained state in individual patients to guide therapy. Candidate markers (monocyte glycolytic flux, H3K4me3 at IL1B promoters, exosomal miRNA panels) require prospective validation (86, 87, 111). Second, distinguishing protective from pathological training is difficult—over−suppression may increase infection risk or impair healing, so therapies must be reversible or tissue−targeted (107, 112, 113). Third, targeting central training at the HSPC level carries theoretical risks of clonal selection, particularly in older patients with CHIP (DNMT3A/TET2) mutations (90, 92, 93). Fourth, traditional 6− to 12−week flare trials are too short to capture the benefit of reprogramming therapies, which require months to consolidate (**Figure 6**) (114, 115).

### 6.3 Key challenges in clinical translation

Despite these diverse therapeutic options, several fundamental challenges remain for translating trained immunity modulation into clinical gout practice. First, no validated biomarkers currently quantify the trained state in individual patients to guide therapy. Candidate markers — monocyte glycolytic flux, H3K4me3 at IL1B promoters, exosomal miRNA panels — require prospective validation (86, 87, 111). Second, distinguishing protective from pathological training is difficult. Over−suppression may increase infection risk or impair tissue healing, so therapies must be reversible or tissue−targeted to preserve normal host defence (107, 112, 113). Third, targeting central training at the HSPC level carries theoretical risks of clonal selection, particularly in older patients with CHIP (DNMT3A/TET2) mutations, which could paradoxically amplify pro−inflammatory clones (90, 92, 93). Fourth, traditional 6− to 12−week flare trials are too short to capture the benefit of reprogramming therapies, which require months to consolidate epigenetic remodelling and reduce long−term flare risk (**Figure 6**) (114, 115).

## 7 Discussion

**Figure 7:**
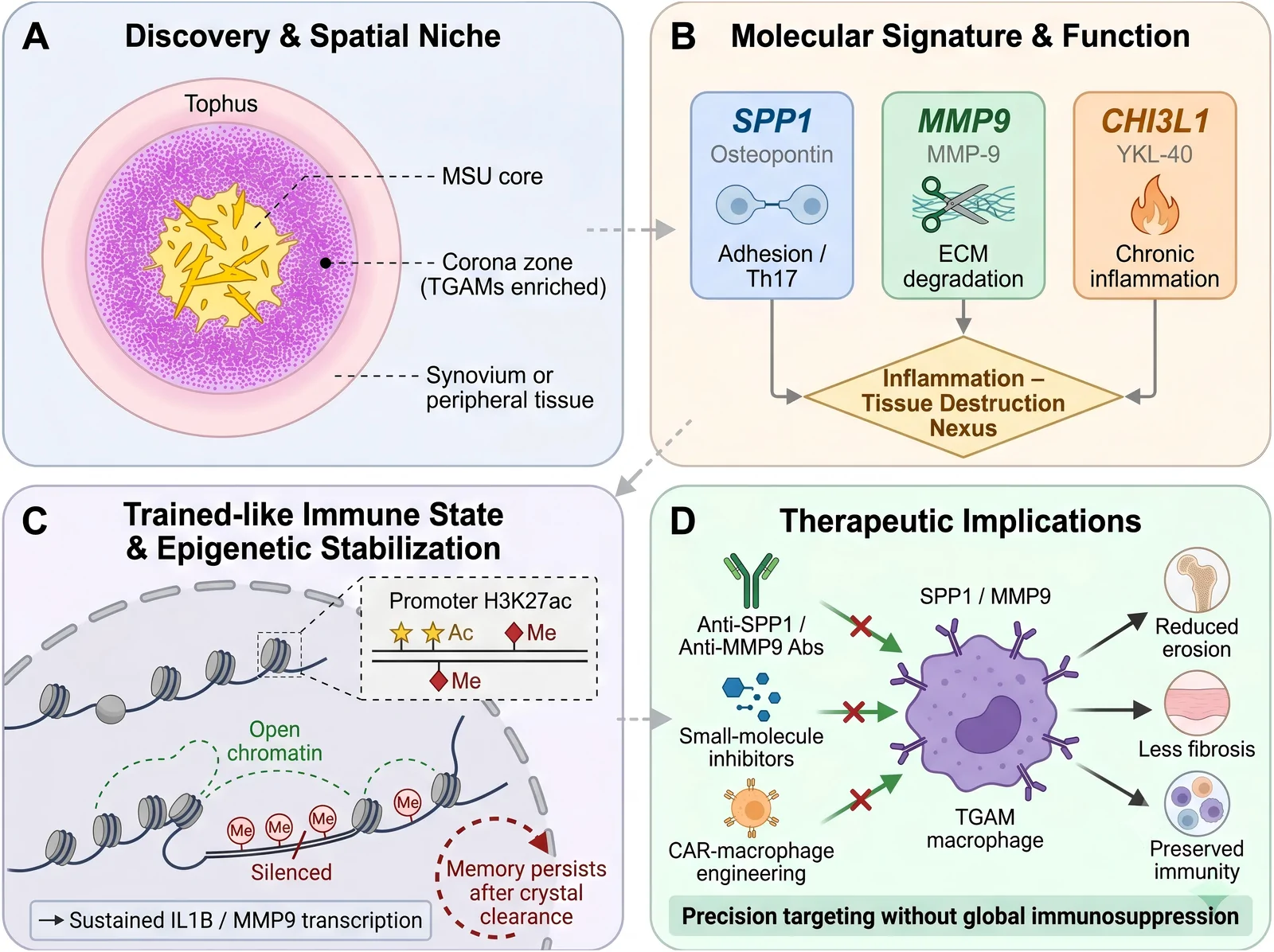
Visual model of systemic inflammation coupling gout to atherosclerosis. **(A)** joint-level MSU crystal deposition and macrophage training; **(B)** release of trained monocytes into circulation; **(B)** bone marrow training of HSPCs; **(D)** infiltration of trained myeloid cells into atherosclerotic plaques.

The trained immunity framework provides a unifying mechanistic explanation for four long−standing clinical paradoxes in gout. Importantly, the mechanistic components of trained immunity—metabolic rewiring and epigenetic modifications—are not unique to innate immune memory; similar changes occur in adaptive lymphocytes. The defining feature of trained immunity lies in its persistence and transmission through hematopoietic stem and progenitor cells (HSPCs), which enables functional memory that outlasts the lifespan of individual myeloid cells and perpetuates a hyper−responsive state even during intercritical periods. Accumulating preclinical and clinical evidence supports a revised perspective: gout is not merely a crystal−deposition arthropathy, but a condition shaped in part by maladaptive innate immune memory that drives chronicity and comorbidities. The spatiotemporal immune gradient framework explains: (i) why only 10–15% of hyperuricemic individuals develop gout—those with permissive epigenetic landscapes or CHIP mutations (DNMT3A/TET2) are primed for hyper−responsiveness, while others maintain epigenetic restraint (90, 116–119); (ii) why flares occur despite serum urate control—stable H3K4me3/H3K27ac and DNA hypomethylation at IL1B/NLRP3 loci persist independently of ongoing crystallisation (120–122); (iii) why cardiovascular risk remains elevated after urate normalisation—systemically trained monocytes infiltrate atherosclerotic plaques, sustaining vascular inflammation without requiring local coronary MSU deposition (83, 84, 123, 124); and (iv)why ageing amplifies both gout and cardiovascular risk—accumulating CHIP mutations may progressively dismantle the DNMT3A–TET2 epigenetic brakes on myeloid inflammation, although direct evidence for a trained−immunity mechanism in patients is currently indirect (**Figure 7**) (90, 92, 93).

Before translating this framework into therapeutic strategies, it is essential to critically appraise the strength of the underlying evidence. As noted throughout this review, much of the data linking trained immunity to gout pathogenesis are derived from ex vivo models using supraphysiological urate concentrations or MSU crystal stimulation of healthy donor cells, rather than from direct longitudinal tracking of trained phenotypes in gout patients. While these mechanistic studies provide compelling biological plausibility and have identified candidate pathways—including JNK–AP−1 signalling, mTOR–HIF−1α−driven metabolic rewiring, and DNA methylation changes—they do not by themselves constitute definitive proof that trained immunity drives disease onset, chronicity, or flares in humans. The heterogeneity of patient responses, the influence of genetic background (including CHIP mutations), and the context−dependent effects of soluble versus crystalline urate further complicate direct extrapolation. Prospective cohort studies with serial functional and epigenetic profiling of circulating monocytes from hyperuricemic individuals who later develop gout are critically needed to establish causality and to define the precise contribution of trained immunity to the clinical trajectory of gout (**Table 4**).

**Table 4:** Candidate biomarkers for quantifying the trained immunity state in gout patients.

Biomarker	Category/sample type	Detection method
DNA methylation signatures at specific CpG loci(e.g.,IL1B,TNF,NLRP3promoters)	Epigenetic/whole blood or isolated monocytes	Bisulfite sequencing, methylation-specific PCR, or array-based profiling
Glycolytic capacity of circulating monocytes	Metabolic/isolated monocytes	Seahorse extracellular flux analysis (ECAR measurement)
Cytokine hyper-responsiveness assay	Functional/whole blood or isolated cells	Ex vivo stimulation with LPS or MSU crystals; measure IL-1β, TNF, IL-6 by ELISA or multiplex
Circulating CHIP mutations(e.g.,DNMT3A,TET2,ASXL1)	Genetic/plasma cell-free DNA or peripheral blood cells	Error-corrected sequencing (VAF ≥0.5%)
Plasma TNFSF14 (LIGHT)	Protein/plasma or serum	ELISA or multiplex immunoassay
Serum CCL18	Protein/serum	ELISA
Exosomal microRNA panels(e.g., miR-155/miR-146a ratio)	Exosomal RNA/plasma or serum	qPCR, small RNA sequencing

CpG, 5’−C−phosphate−G−3’ sites; IL1B, interleukin−1 beta gene; TNF, tumour necrosis factor gene; NLRP3, NOD−, LRR− and pyrin domain−containing protein 3 gene; PCR, polymerase chain reaction; ECAR, extracellular acidification rate; ELISA, enzyme−linked immunosorbent assay; CHIP, clonal haematopoiesis of indeterminate potential; DNMT3A, DNA methyltransferase 3A; TET2, ten−eleven translocation 2; ASXL1, additional sex combs−like 1; VAF, variant allele fraction; TNFSF14, tumour necrosis factor superfamily member 14; CCL18, C−C motif chemokine ligand 18; qPCR, quantitative polymerase chain reaction.

Collectively, these insights call for a broader therapeutic perspective that moves beyond the inflammasome−centric view. Future strategies should consider targeting inflammasome−independent pathways, including JNK–AP−1 signalling and DNMT3A−mediated metabolic adaptation, which may offer complementary benefits in modulating the trained state without completely abrogating IL−1β−dependent host defence. Such an approach could help preserve essential innate immune functions while specifically dampening the maladaptive memory that drives chronic gout.

It is important to acknowledge that much of the current evidence linking trained immunity to gout derives from ex vivo models using supraphysiological urate concentrations or MSU crystals stimulation of healthy donor monocytes, rather than from direct longitudinal tracking of trained phenotypes in gout patients. While these mechanistic studies provide compelling biological plausibility, they do not constitute definitive proof that trained immunity drives gout pathogenesis in humans. Prospective cohort studies with serial functional and epigenetic profiling of circulating monocytes from hyperuricemic individuals who later develop gout are critically needed to establish causality.

As detailed above (Section 6 and **Table 3**), translating this framework faces hurdles including biomarker validation, the need to reprogramme rather than ablate innate memory, clonal selection risks in CHIP−positive patients, and trial designs sufficiently long to capture epigenetic consolidation.

Emerging frontiers promise to refine patient stratification and identify novel nodes for intervention. First, single−cell multi−omics and spatial transcriptomics of tophi and atherosclerotic plaques will enable high-resolution mapping of trained immune cell subsets and their epigenetic states in human tissues. Second, AI−driven integration of multi-omic, clinical, and imaging data may identify composite biomarkers of trained immunity that outperform single molecular markers. Third, modulation of the gut–joint–vascular axis via butyrate-producing probiotics or dietary interventions represents a holistic approach to fine-tune myeloid reprogramming (59, 125, 126).

Fourth, sex dimorphism in trained immunity represents an underexplored but clinically critical research direction. Gout exhibits a striking male predominance, with premenopausal women having substantially lower disease risk — a difference partially attributed to oestrogen’s uricosuric and direct anti-inflammatory effects. Emerging preclinical evidence indicates that oestrogen can modulate multiple core pathways of trained immunity: it suppresses mTOR/HIF-1α-mediated glycolytic reprogramming in myeloid cells, upregulates DNA methyltransferase expression to counteract pro-inflammatory hypomethylation, and directly inhibits NLRP3 inflammasome assembly. These effects suggest that female sex hormones may raise the threshold for establishing pathological trained immunity in response to urate exposure, contributing to the lower gout incidence in premenopausal women. Prospective studies stratified by sex and hormonal status are needed to dissect the interactions between sex hormones, epigenetic reprogramming, and gout progression, which may inform sex-specific risk stratification and therapeutic strategies (93, 94).

Limitations include the debated extension of trained immunity to adaptive lymphocytes, the cross−sectional nature of most human epigenetic studies, and the predominance of preclinical findings awaiting clinical validation. In conclusion, the trained immunity paradigm reclassifies gout as a disease of innate immune memory. This shifts the therapeutic goal from suppressing flares to modifying disease. Ultimately, therapy should reset the epigenetically primed myeloid state to prevent disease progression. Success will depend on validated biomarkers, careful patient selection (especially regarding CHIP), and trial designs that capture long−term modification rather than short−term symptom control. Critically, the field must move beyond indirect evidence from ex vivo models to establish the precise contribution of trained immunity to gout through prospective human studies, including serial immune phenotyping of high−risk hyperuricemic individuals, integration of multi−omics with clinical outcomes, and well−designed interventional trials with epigenetic and functional endpoints. Only then can the trained immunity paradigm be rigorously translated into clinical practice (13, 22, 127, 128).
